# Clinical Proteomics

**DOI:** 10.1155/2012/641491

**Published:** 2012-08-15

**Authors:** Ákos Végvári, Tadashi Kondo, John G. Marshall

**Affiliations:** ^1^Clinical Protein Science and Imaging, Lund University, 221 84 Lund, Sweden; ^2^Division of Pharmacoproteomics, National Cancer Center Research Institute, Tokyo, Japan; ^3^Department of Chemistry and Biology, Ryerson University, Toronto, ON, Canada M5B 2K3

In a rapidly developing technological environment, including advances of mass spectrometric platforms, proteomic applications gain ever-increasing attentions in particularly linked to diseases, offering new means to improve the state-of-the-art in diagnosis and therapy [1]. However, clinical proteomics should not be considered as a collection of studies dealing with analyses of clinical samples but rather studies that can raise clinically relevant questions. As such, clinical proteomics systematically employs proteomic technologies, laboratory medicine, bioinformatics in order to identify protein patterns of diseases comprehensively that can lead to improved patient care and public health for the benefit of better assessment of prevention of disease, detection and diagnosis of disease, selection of personalized therapy, and monitoring of treatment response.

In this Special Issue, we present a small set of research and review articles contributing to the field of clinical proteomics. Today, biomarker discovery is certainly one of the most important and widely investigated areas. Although, current routine analyses in clinical chemistry include more than 200 proteins being analyzed in blood samples and an addition number of protein markers that are used for flow cytometry and antigens, none of these biomarkers have been originating from proteomics [2]. Furthermore, quantification of proteins with clinical importance is largely performed by immunoreaction-based assays (e.g., ELISA), where the available methodologies are numerous. However, modern mass spectrometry developments have already brought sufficient power, which are complementary to or even competitors of immunoreaction-based technologies in biomarker research [3].

The essential advantage of the proteomic approach is the potential to identify and quantify multiple biomarkers simultaneously in the large parts of the human proteome, providing an overall view of differential expression of proteins in blood or tissue. Ultimately, this may lead to the distinction of disease phenotypes, which has a growing importance in cancer research, where the personalized treatment of patients offer more sufficient targeted therapy, and thus can ease the burden of healthcare.

Tissue samples, well characterized by pathologist, are particularly useful subjects for such comparative proteomic analyses, which may reveal new insights to the heterogeneity of diseases like cancer. Our research experiences indicate that deep proteomic analyses of these clinically valuable samples require thorough preparation procedures, which are currently a major focus of developments. Novel and effective preparation methods in combination with archived biobanking material, such as formalin-fixed and paraffin-embedded tissues, will be beneficial for future improvements [4].

Lastly, we would like to express our appreciation to the authors of this Special Issue for their contributions and the staff of International Journal of Proteomics for their meticulous handling of the articles. We strongly hope that the readers this Special Issue can provide novel and stimulating data and views of clinical proteomics.

*Ákos Végvári* *Ákos Végvári* *Tadashi Kondo* *Tadashi Kondo* *John G. Marshall* *John G. Marshall*
